# Understanding Quorum-Sensing and Biofilm Forming in Anaerobic Bacterial Communities

**DOI:** 10.3390/ijms252312808

**Published:** 2024-11-28

**Authors:** Kinga Markowska, Ksenia Szymanek-Majchrzak, Hanna Pituch, Anna Majewska

**Affiliations:** Department of Medical Microbiology, Medical University of Warsaw, 5 Chalubinski Str., 02-004 Warsaw, Poland

**Keywords:** anaerobic bacteria, autoinducers, biofilm, quorum sensing, quorum quenching

## Abstract

Biofilms are complex, highly organized structures formed by microorganisms, with functional cell arrangements that allow for intricate communication. Severe clinical challenges occur when anaerobic bacterial species establish long-lasting infections, especially those involving biofilms. These infections can occur in device-related settings (e.g., implants) as well as in non-device-related conditions (e.g., inflammatory bowel disease). Within biofilms, bacterial cells communicate by producing and detecting extracellular signals, particularly through specific small signaling molecules known as autoinducers. These quorum-sensing signals are crucial in all steps of biofilm formation: initial adhesion, maturation, and dispersion, triggering gene expression that coordinates bacterial virulence factors, stimulates immune responses in host tissues, and contributes to antibiotic resistance development. Within anaerobic biofilms, bacteria communicate via quorum-sensing molecules such as N-Acyl homoserine lactones (AHLs), autoinducer-2 (AI-2), and antimicrobial molecules (autoinducing peptides, AIPs). To effectively combat pathogenic biofilms, understanding biofilm formation mechanisms and bacterial interactions is essential. The strategy to disrupt quorum sensing, termed quorum quenching, involves methods like inactivating or enzymatically degrading signaling molecules, competing with signaling molecules for binding sites, or noncompetitively binding to receptors, and blocking signal transduction pathways. In this review, we comprehensively analyzed the fundamental molecular mechanisms of quorum sensing in biofilms formed by anaerobic bacteria. We also highlight quorum quenching as a promising strategy to manage bacterial infections associated with anaerobic bacterial biofilms.

## 1. Introduction

The National Institutes of Health (NIH) has shown that 60–80% of all microbial infections are associated with biofilm formation [1]. The first description of surface-attached, structured microbial communities, later referred to as biofilm, came from the Dutch scientist Antoni van Leeuwenhoek, who in 1683 observed the aggregated bacteria in plaque on the teeth and, particles scraped from the tongue [2,3]. In the 1930s, biofilm was observed in the aquatic environment. In medicine, the first association between the etiology of chronic infection and aggregates of bacteria was evidenced during routine microscopic examination of Gram-stained sputum in the early 1970s by Nils Høiby. The sample was taken from cystic fibrosis patients with persistent *Pseudomonas aeruginosa* infection. In 1980, Costerton and co-workers published the image of *P. aeruginosa* microcolonies in a lung of a patient (post-mortem) with cystic fibrosis made by electron microscope [4]. The definition of biofilm has evolved over the last decades. The first medical report in which the word ‘biofilm’ was used was published in 1981 by dentists from the University of Lund, Sweden which showed that biofilm forms on all solid surfaces in the oral cavity [2,5,6]. The improved identification of anaerobic bacteria (by applying matrix-assisted laser desorption/ionization time-of-flight mass spectrometry; MALDI-TOF MS and 16S rRNA genes cluster sequencing) in clinical specimens has raised awareness of the fact that anaerobes are involved in infections in almost every part of the body [7,8,9]. It is now known that certain anaerobes can form mono-, dual- and even multi-species biofilms, although this property and its impact on infectious disease remain to be studied in more detail.

Under natural, physiological conditions, bacteria form biofilms on human tissues such as the skin, mucosal surfaces of the respiratory and digestive tracts, and the female genital tract. These biofilms have a protective role, preventing the emergence of infections. However, under certain circumstances, such as disruption of the continuity of the skin and mucous membranes due to trauma, diabetic foot or ulcerative colitis, peripheral vascular disease, or peripheral neuropathy, these niches can be overgrown by biofilm-forming pathogenic organisms. Biofilm-associated infections are usually chronic, can recur, and are, therefore, very difficult to control [10,11,12]. A thorough understanding of the clinical impact of biofilms formed by anaerobic organisms is not presented in the literature, most likely due to the difficulty and technical knowledge required to culture anaerobic organisms. Understanding the mechanism of biofilm formation and inter-bacterial interactions appears to be crucial for the development of strategies to combat pathogenic biofilms.

In this paper, we have reviewed the scientific literature with a primary focus on the involvement of anaerobic biofilms in infectious diseases. The main aim is to summarize the molecular mechanisms of cell-to-cell communication, known as quorum sensing (QS), within biofilms formed by anaerobic bacteria. This review also points to the inhibition of QS systems, called quorum quenching (QQ), which is now expected as a promising strategy to combat bacterial biofilm-associated infections.

## 2. Methods

**Figure 1:**
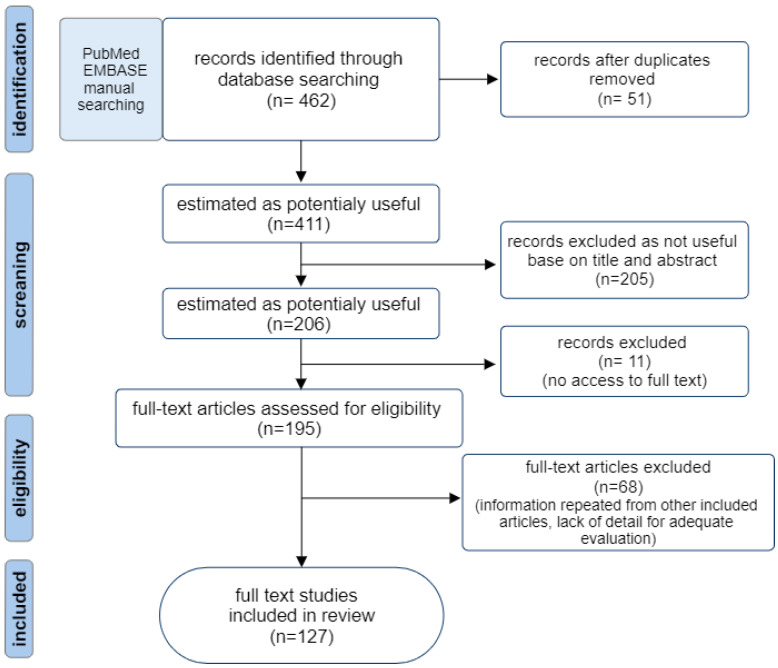
Flow diagram of the literature search strategy.

The preliminary search was conducted to identify relevant articles to ensure the validity of the proposed idea and to ensure that we have enough sources to conduct the analysis. An electronic search was performed querying the following databases MEDLINE (accessed through PubMed), and EMBASE from January 1990 through October 2024. A manual search of publications consistent with the topic was also performed. The search strategy was constructed to include free-text terms in the title and abstract and any appropriate subject indexing (quorum sensing, quorum quenching, autoinducer, acyl homoserine lactone, signaling molecules, sensing molecule, *Fusobacterium*, *Prevotella*, *Porphyromonas*, *Bacteroides*, *Clostridium*, *Clostridioides*, *Cutibacterium*, *Propionibacterium*, anaerobic bacteria, biofilm) with Boolean operators. Full-text articles written in English were included in subsequent screening. For removing the duplicates, the automated systematic search deduplicator (ASySD) was used. The two researchers evaluated articles individually using the same searching criteria with a particular focus on relevance to the scope of this review. The search results were compared, and a list of eligible publications was compiled. The classified studies were thoroughly reviewed, and relevant data were extracted. The organization of the literature search is shown in the diagram (Figure 1).

## 3. Biofilms and Their Medical Impacts

**Figure 2:**
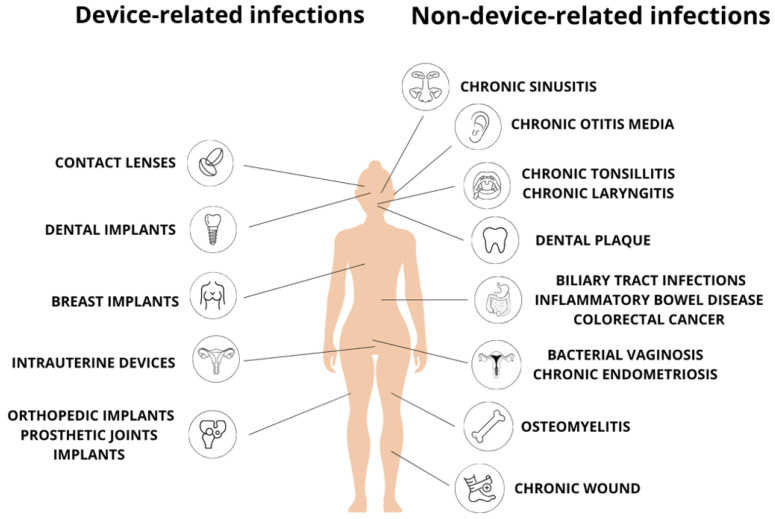
Location of infections associated with biofilm formed by anaerobic bacteria.

Biofilms are sessile microbial communities and have been described as coherent clusters (microcolonies) of bacterial cells embedded in an extracellular polymeric substance (EPS). EPS can account for up to 80% of the total volume of biofilms and is composed of polysaccharides (e.g., alginate), proteins (e.g., fibrin), lipids, metal ions, and extracellular DNA (eDNA) [10]. Multispecies biofilm, composed of different microorganisms (e.g., dental biofilm which may consist of up to hundreds of species) is a common phenomenon [11]. Within the biofilm, bacteria alter their own metabolism and protein production, regulate gene expression which can lead to a reduced cell division rate, and as a result, adapt to environmental anoxia and nutrient limitation. Compared to bacteria in the planktonic (freely suspended) state, biofilm-forming bacteria exhibit much more virulent properties, are better adapted to environmental conditions, and can be highly resistant or tolerant to antimicrobials. Biofilms constitute an optimal environment for horizontal gene transfer thus promoting the spread of bacterial resistance to antimicrobial agents [12,13]. Biofilms that affect human health can be divided into three categories: formed on human tissues, formed on medical devices, and formed on surfaces outside the body. Surfaces in the mouth are easily colonized by bacteria that form a biofilm called dental plaque. The accumulation and maturation of dental plaque on tooth surfaces can lead to inflammatory disease, gingivitis, periodontitis, and irreversible destruction of tooth-supporting tissues, peri-implantitis [11,14]. Anaerobes colonize the oral cavity and have a potential tumor-promoting effect (oral squamous cell carcinoma). Currently, there are three periodontopathogens involved in carcinogenesis. Two of them are anaerobic bacteria, *Porphyromonas gingivalis*, and *Fusobacterium nucleatum*, the third is an aerobic organism *Treponema denticola* [15,16,17]. Approximately 10% to 40% of all sinusitis cases are the result of an odontogenic process. The understanding of the etiology and pathogenesis of chronic sinusitis is still incomplete. Correct diagnosis requires aspiration of maxillary sinus secretions and/or ethmoid sinuses by endoscopy, which is not a standard procedure. Failure to respond to first-line therapy may be associated not only with the emergence of resistant aerobic strains but also with anaerobic bacterial infections [18]. There is growing evidence that the oral pathogen *F. nucleatum* is involved in the progression of an increasing number of tumor types, including colorectal, pancreatic, esophageal, and breast cancers [19]. Moreover, anaerobic bacteria (mainly *Bacteroides* spp., *Peptostreptococcus* spp., *Fusobacterium* spp., *Clostridium* spp. as well as *Cutibacterium acnes*) can cause septic arthritis and osteomyelitis [20]. Commensal bacterium *C. acnes* can be responsible for up to 10% of bacterial prosthesis joint infections [21]. It is worth noting that anaerobes may accompany aerobic bacteria (mainly facultative streptococci and gram-negative bacilli) in mixed infections. Under such conditions, it is not clear which organism or organisms are the main infective agents of bone infection or whether there is a synergistic mechanism of infection [22]. Anaerobes are also involved in pelvic inflammatory diseases and endometritis [23,24]. Numerous studies have confirmed the contribution of *Bacteroides fragilis* in inflammatory bowel disease (IBD), Crohn’s disease, and colitis ulcerosa. Enterotoxigenic *B. fragilis* (ETBF) strains secrete a 20 kDa pro-inflammatory zinc-dependent metalloprotease that can stimulate high expression of host interleukin-17 and increase the permeability of intestinal epithelial cells, resulting in enhanced internalization of various intestinal bacteria [25]. In ulcerative colitis, some species classified as *Bacteroidetes* correlate with disease activity [26]. The locations of infections associated with biofilms formation are shown in Figure 2.

Anaerobic biofilm can be formed on medical devices, including the following:

- orthopedic implants [21,27,28],
- dental implants [11,27,29],
- breast implants used in both postmastectomy breast reconstruction and cosmetic surgical procedures [27,30],
- contact lenses [27,29],
- intrauterine devices, e.g., as long-term contraception methods [27].

The biofilms can be formed also on surfaces outside the body, mainly in water distribution systems, and if not systematically removed, can be a source of infection, including nosocomial infections [31].

However, the contribution of anaerobes to biofilm formation is becoming better understood, and biological mechanisms of quorum-sensing signaling molecules have yet to be adequately analyzed. A comprehensive understanding of the microbial signaling cascade provides a basis for interpreting microbial behavior in infection-related diseases and, in perspective, for using molecular modeling as a step in the search for new compounds with potential antibiofilm activity.

**Figure 3:**
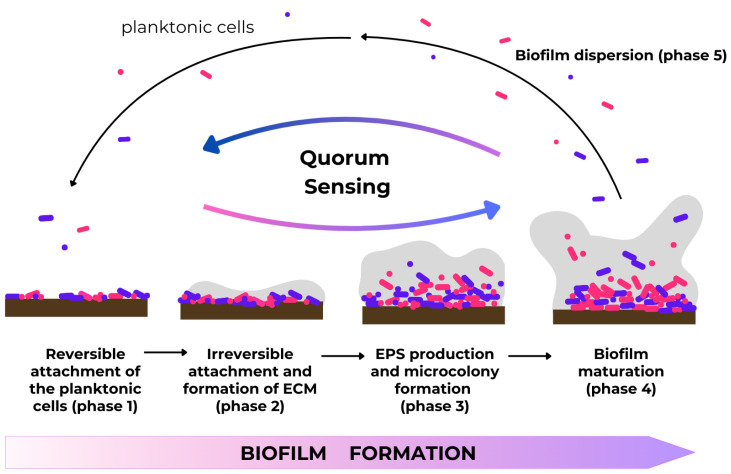
Schematic of the five main stages of biofilm formation.

The formation of bacterial biofilms is a complex phenomenon, determined by many physical, chemical, and biological processes. Biofilm architecture is heterogeneous. It is constantly changing in both time and space due to external and internal factors. In the process of biofilm formation, phases such as reversible attachment of the planktonic cells (1), irreversible attachment and formation of extracellular matrix (ECM) (2), EPS production and microcolony formation (3), biofilm maturation (4), and final dispersion (5) have been identified (Figure 3) [32].

Phase 1 and Phase 2: Attachment. The initial phase is the reversible attachment of the microorganism to a solid surface using gravitational, electrostatic interactions, and van der Waals forces or bacterial structures such as flagella or pili. In this phase, cohesion also occurs. The next step is the irreversible binding of the bacteria to the surface and the production of an extracellular matrix. ECM promotes biochemical and physiological changes in the biofilm matrix which ensures structural integrity; stability; access to nutrients, metabolites, or signaling substances; and protection from adverse external factors (bactericides), desiccation, and host defense factors. During the irreversible attachment phase, bacterial cells interact with the surface using adhesins and lipopolysaccharide (LPS). This interaction causes changes in the surface properties including small damages and hydrophobicity decreasing. The binding intensity depends primarily on the species and the number of microorganisms adhering to the surface as well as the physicochemical characteristics of the surface. The speed and extent of biofilm growth depend, among other factors, on liquid flow rate, Brownian motion, nutrient availability, iron availability, pH, osmolality, oxygen concentration, concentration of antibacterial drugs, and ambient temperature. In such specific environment conditions (pH and oxygen gradients, co-adhesion), microorganisms synthesize, and release signaling molecules, communicate with each other, and change the surrounding microenvironment. The composition and structure of the biofilm matrix can evolve over time [32,33,34,35].

Phase 3: Microcolony formation. This phase is characterized by the synthesis of extracellular polymers (soluble and insoluble glucans, fructans, and heteropolymers). The matrix is biologically active, with channels retaining water, nutrients, and enzymes within the biofilm structure. In addition, the presence of certain bacteria creates an ecological niche for other microorganisms, enabling them to survive in the new favorable conditions [36,37].

Phase 4: Biofilm maturation and bacterial succession. The formation of a mature biofilm is associated with increased cell density and a reduction in the growth rate of specific bacteria. At this stage, interactions between microorganisms and host play the most important role not only in the formation of a mature biofilm structure but also in the separation of bacterial species/cells from such a formed structure. Bacteria can ‘sense’ specific environmental changes and induce the genes associated with active detachment [34]. One example is *Prevotella loescheii* (renamed as *Hoylesella loescheii*), which produces proteases that hydrolyze its adhesins, which are responsible for co-aggregation with *Streptococcus mitis* [36].

Phase 5: Dispersion. Finally, to avoid overgrowth, which would reduce the bacteria’s access to vital nutrients and lead to the accumulation of harmful waste products, bacterial cells detach from the biofilm and migrate as planktonic forms. In this way, microorganisms can spread throughout the human body. Therefore, a mature biofilm structure results from a balance between adhesion, growth, and microorganism removal [32,34,36].

Slow-growing cells in a biofilm phenotype are generally less sensitive or even resistant to antibiotics, antiseptics, and both innate and acquired immune responses, compared to planktonic cells. The extracellular matrix interacts with the environment, e.g., by attaching anaerobic or anaerobic-aerobic biofilms to human tissues (mucous membranes and damaged skin) or artificial materials. Within the biofilm, the individual bacteria communicate with each other by finely tuned molecular processes defined as quorum sensing [32,38].

## 4. Quorum Sensing and Quorum-Sensing Molecules (QSMs)

Biofilms are characterized by a high degree of structural and functional bacterial organization. Cell-to-cell communication within the biofilm is based on the generation and sensing of extracellular signals and the secretion of specific small signaling molecules called autoinducers (AIs). QS signals play a role in the biofilm development and dispersal. The concentration of signaling molecules is positively correlated with microbial population density.

When signaling molecule concentration reaches a certain threshold, they bind to intracellular receptors to activate target gene expression coordinating inter- and intra-population physiological behavior, including facilitating adaptation to the environment, bacterial motility, coordination of virulence factor expression, sporulation, and adhesion [39,40,41,42]. Quorum-sensing systems consist of a membrane-bound histidine sensor kinase and a cytoplasmic response regulator, which can function as a transcription factor [43]. In addition to their effects on the bacterial life cycle, quorum-sensing molecules (QSMs) also induce immune responses in host tissues [44,45] and have an impact on the development of antibiotic resistance [46,47].

In anaerobic bacteria, AIs are classified into three major classes:

- N-Acyl homoserine lactones (AHL, autoinducer type-1, AI-1) specific to Gram-negative bacteria,
- autoinducer-2 molecules (AI-2), consisting of 4,5-dihydroxy-2,3-pentandedione (DPD) derivatives in both Gram-negative and Gram-positive bacteria,
- autoinducing peptides (AIPs), specific to Gram-positive bacteria [46,48].

**Figure 4:**
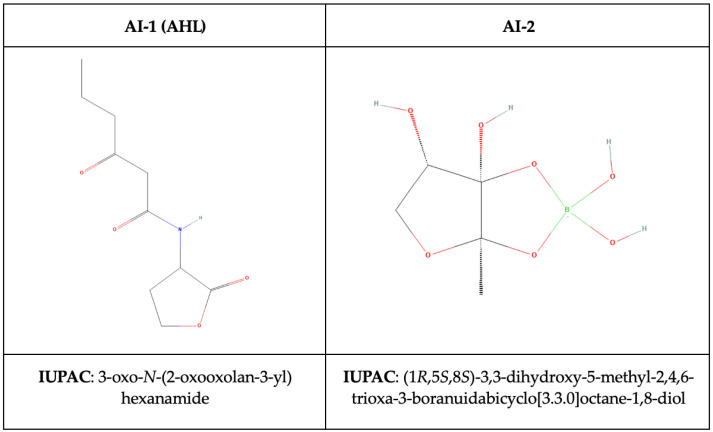
Chemical structure of the signaling molecules: type 1 (AI-1, AHL) and type 2 (AI-2) (found in *Vibrio harveyi*) [49].

The chemical structure of the compounds AI-1 and AI-2 are shown in Figure 4.

### 4.1. N-Acyl-Homoserine Lactones (AHLs)

**Figure 5:**
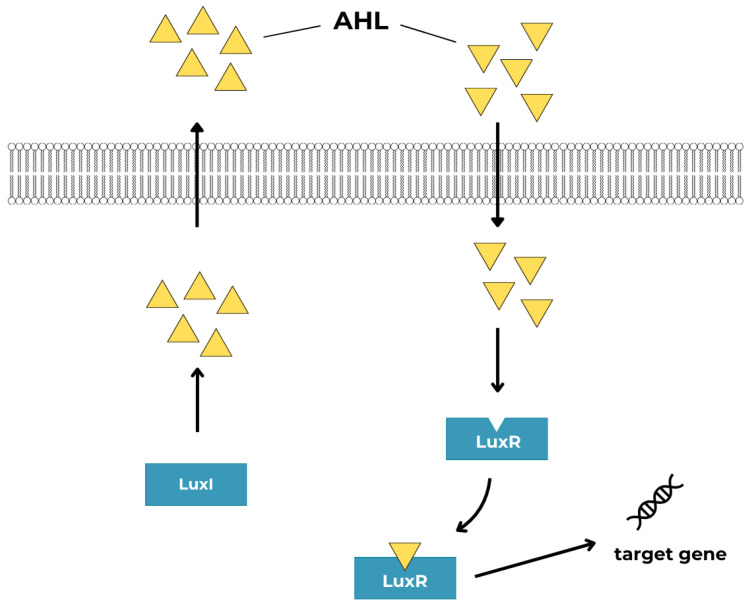
Schematic diagram of AHL signaling molecules regulating the QS system through the LuxI/LuxR pathway.

The type I autoinducers (AI-1) are represented by N-Acyl-homoserine lactones (AHLs) first described in the aerobic pathogen, *Pseudomonas aeruginosa* [50]. AHLs are a family of small diffusible signaling molecules, produced by a range of Gram-negative environmental and pathogenic bacteria [51]. AHLs consist of a hydrophilic homoserine lactone ring (S-adenosylmethionine) and hydrophobic acyl side chain (of varying length; short-chain AHLs with C4–C8 and long-chain AHLs with C10–C20). The degree of hydrophobicity increases with the length of the acyl side chain. The diversity of AHL molecular structures is caused by differences in the R group and the substituent group in the acyl chain [52]. AHLs are synthesized in the cell by acyl-homoserine lactone synthase enzyme (LuxI). Gene *luxI* is expressed at the basal level at low population density. The concentration of an AHL increases along with the growth of the bacterial cell population. AHL passively diffuses or passes with the support of transport proteins through the cell membrane down a gradient to the environment. When the threshold is reached, the AHL signal returns to the cell and binds to the cognate LuxR receptor. Signaling works through the LuxI/LuxR system [50,52,53,54]. The LuxR/AHL protein complex binds to promoter DNA regions and regulates the transcription of QS-regulated genes. Thus, it can regulate, e.g., carbon, nitrogen, and sulfur metabolism, allowing bacteria to quickly adapt to extreme environmental conditions [52]. Figure 5 shows a schematic presentation of an AHL-mediated QS system.

The production of AHLs can be determined by factors including pH, substrate concentration, and a carbon/nitrogen (C/N) ratio. AHLs have been detected in saliva and sputum samples [51], and in the human gastrointestinal tract [55]. The effect of AHL on infection processes involving anaerobic bacteria like *Bacteroides* spp. and *Porphyromonas* spp. has been described so far. Some bacteria, such as *Bacteroides* spp., have no capacity to synthesize AHLs due to the lack of LuxI homologs. Bacteria that have only LuxR homologs are called LuxR solo or LuxR orphans and, curiously, they can sense AHL from other bacteria present in the biofilm [52,56,57]. *B. fragilis* can respond to the presence of exogenous homoserine lactones and thereby modulate the bacterial society and regulate gene expression [52]. In the *B. fragilis* genome, LuxR orthologs (so far nine putative QS gene homologs of *luxR*) were identified. Pumbwe et al. reported an over-representation of *luxR* genes (luxR5-luxR9) in *B. fragilis* cells grown in a medium enriched with AHL (N-hexanoyl homoserine lactone C6-HSL) [58]. According to Grellier et al., the expression of *luxR* from *Bacteroides* spp. could be linked to inflammatory bowel disease-associated dysbiosis [59].

Interestingly, AHL regulates its own synthase and receptor genes in a positive feedback loop. This phenomenon has been described in other pathogens (e.g., aerobic bacteria, *P. aeruginosa*), while there is still a gap in understanding the role of commensal-derived AHL and their impact on the host, in particular on the gut ecosystem [50]. The results demonstrated by Muras and co-workers indicate a potential role of AHLs in the development of dysbiosis related to periodontal diseases. C6-HSL increases the abundance of *Alloprevotella*, *Peptostreptococcus*, and *Prevotella* species in periodontal biofilms. Some members of the *Peptostreptococcus* (*P. micros*) and *Prevotella* (*P. intermedia*, *P. nigrescens*) genera are included in, the so-called, orange-complex associated with periodontitis AHLs and seem to influence the growth and protein expression by a key periodontopathogen, *P. gingivalis*, a member of the red-complex [60,61].

It has been shown that AHLs can specifically affect lactic acid production, disrupt epithelial integrity by activating host cell protease secretion, and reduce the level of proteins responsible for the integrity of the epithelium (occludin and tricellulin). Occludin is one of the factors contributing to the inflammatory process associated with *P. gingivalis.* This is probably due to the activities of bacterial gingipains (involved in the degradation of cytokines) and epithelial matrix metalloproteinases (MMPs), which degrade components of the basal lamina, epithelial cell-cell junctions, and collagen [62]. Results of the study conducted by the Muras team revealed that *P. gingivalis* produced a small quantity of octanoyl-L-homoserine lactone OC8-HSL (0.3 ng/mL). The higher concentration of this molecule was observed in a dual-species biofilm formed with *Streptococcus gordonii* (0.83 ng/mL) or *Streptococcus oralis* (1.4 ng/mL) [51]. Multispecies biofilms composed of both aerobic and anaerobic bacteria are clinically relevant.

All these data strongly support the importance of AHL in the oral biofilm community. However, more studies are needed to identify the key players in AHL-mediated QS processes in plaque formation. The molecular phenomena of AHLs in biofilm formation processes involving anaerobes have not been thoroughly investigated. Most studies have been conducted with environmental bacteria or a small number of aerobically growing human pathogens. Considering the unquestioned role of anaerobes in the pathophysiology of infections, this gap needs to be filled.

### 4.2. Autoinducer-2 (AI-2)

Autoinducer-2 molecules are universal, non-species-specific quorum-sensing signal compounds found in both Gram-negative and Gram-positive bacteria. AI-2’s chemical structure is the same for many bacteria species. AI-2 represents a group of (4S)-4,5-dihydroxy-2,3-pentanedione ([S]-DPD) derivatives produced by a complex process, in turn, involving LuxS (homodimeric metalloenzyme encoded by the gene *luxS*) [63]. In brief, *S*-adenosyl-L-methionine (SAM), a substrate for methyl transfers, is biosynthesized from L-methionine and ATP by methionine adenosyltransferase (MAT), the product of the *metK* gene. S-adenosyl-L-homocysteine (SAH) is produced from SAM through demethylation mediated by methyltransferases (MTases). SAH can be modified through two pathways: either by a single-step conversion to homocysteine (HCY), involving SAH-hydrolase or by a two-step process catalyzed by the Pfs and LuxS enzymes. Nucleosidase Pfs irreversibly cleaves SAH into adenine and SRH. LuxS converts SRH into 4,5-dihydroxy-2,3-pentanedione (DPD) and HCY. DPD undergoes further changes to form the active AI-2 molecule [64]. AI-2s are released from the cells and once AI-2 reaches a critical concentration outside the cell, it is transported back through the membrane channel via the LsrACBD transport system. In the cytoplasm, AI-2 is phosphorylated by bacterial kinase, LsrK. AI-2-P acts as a derepressor of the *lsr* operon by inactivation of LsrR. The AI-2-P degradation by LsrFG was conducted simultaneously.

**Figure 6:**
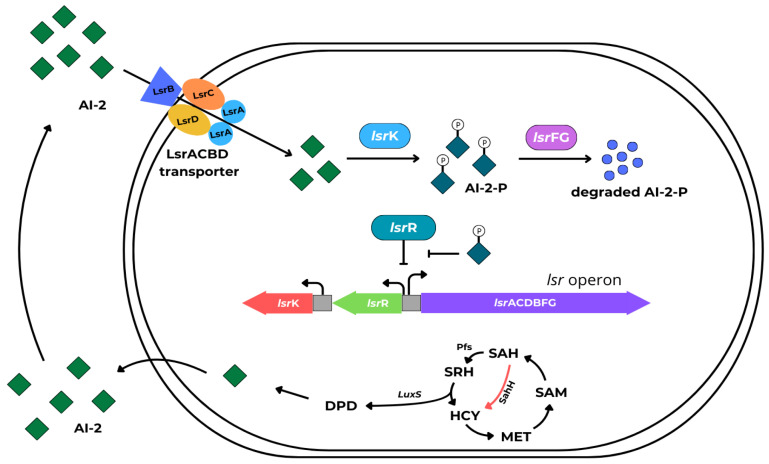
Schematic diagram of AI-2 signaling molecules regulating the QS system.

In these conditions, the processing of DPD is accelerated, promoting the positive regulation of AI-2 QS [42,46,65]. The AI-2-mediated signaling system is shown in Figure 6.

The synthesis of AI-2 or the presence of the gene *luxS* has been detected in many anaerobic pathogenic and commensal rods, such as *Actinomyces naeslundii*, *F. nucleatum*, *P. gingivalis*, *P. intermedia*, *C. acnes*, *Clostridioides difficile*, and oral streptococci [46,66,67,68,69].

Although it is extensively studied, *luxS*-dependent AI-2 signaling remains not entirely understood. In the first decade of the 20th century, the impact of autoinducers on biofilm processes was mainly explored by phenotypic studies with *luxS* mutant strains [70]. Nowadays, transcriptomic [69,71] and proteomics analyses [67,72] are also used.

Inactivation of the *luxS* gene influences a variety of processes, such as type III secretion system, cell motility, biofilm formation, production and release of virulence factors, and antibiotic production [46,67,73]. From a clinical point of view, AI-2 may mediate bacterial colonization of the intestinal tract, altering the composition of intestinal bacteria and modulating the host immune response, leading to inflammatory bowel disease. AI-2 participates in the formation of the subgingival biofilm and affects the virulence of periodontal pathogens [46,65,66]. In *P. gingivalis*, which belongs to the red-complex periodontopathogens, LuxS/AI-2 signaling is involved in the regulation of hemin acquisition and growth under hemin-limited conditions, as well as the expression of proteases and stress-related genes [74,75,76,77,78]. AI-2 plays also a crucial role in the biofilm formation by orange-complex bacteria [79]. AI-2 secreted by *F. nucleatum* promotes co-aggregation and expression of adhesion molecules in *P. gingivalis*, *T. denticola,* and *Tannerella forsythia* and thereby *the* formation of mixed biofilms [47,70]. As demonstrated by Kolenbrander et al., the synthesis and activity of AI-2 are lower in commensal bacteria than in periodontal pathogens [80]. The accumulation of AI-2 could serve as evidence or contribute to the transition from a commensal to a pathogenic biofilm [54]. AI-2 synthetase can affect the host’s pro-inflammatory response [47]. Wu and coworkers showed that *F. nucleatum* AI-2 activates multiple signaling pathways in macrophages. The most significantly upregulated protein is the tumor necrosis factor ligand superfamily member 9 (TNFSF9), which participates in regulating immune cell infiltration in pancreatic adenocarcinoma. Thus, AI-2 may be a novel point of study for the association between bacteria and cancer [72,81]. AI-2 is also involved in signaling among *C. difficile* cells. *C. difficile* QS is involved in sporulation and toxin production, as well as activation of the flagella and colonization of the intestinal tract [42]. In a study conducted by Slater et al., a mutant defective in the *luxS* strain did not produce AI-2 and could not form a biofilm in vitro. In addition, it is hypothesized that *C. difficile* LuxS/AI-2 may use different mechanisms to mediate the formation of single-species and mixed-species communities [69].

In *Clostridium perfringens*, the AI-2 system enhances the expression of toxins in the mid-exponential period [82]. AI-2 is used by *C. acnes* to the regulation of virulence of biofilms and has an impact on the stimulation of the immune system and inflammation [83].

Table 1 shows the best-understood processes regulated by AI-2 in biofilm formed by anaerobic bacteria.

### 4.3. Autoinducing Peptides (AIPs)

**Figure 7:**
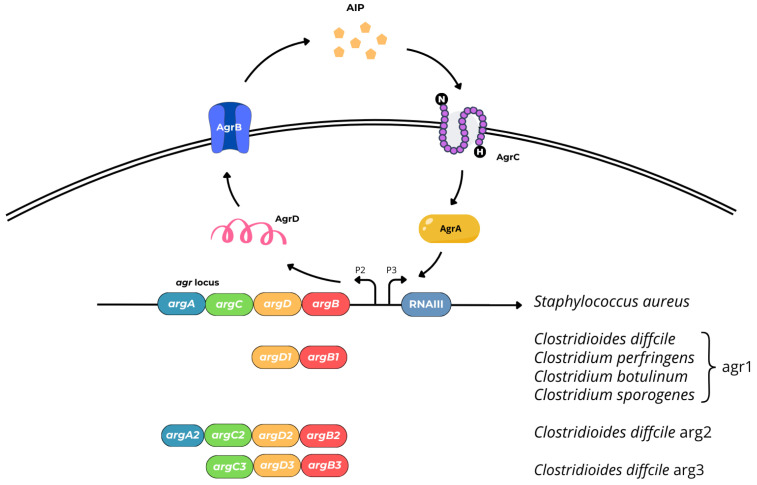
Genetic structure and organization of AIP-mediated signaling Agr system in *S. aureus*, *C. difficile*, and *Clostridium* spp.

Autoinducing peptides (AIPs), are cyclic oligopeptides that contain a thiolactone ring formed by condensation of the C-terminal carboxyl group and the sulfhydryl group of an internal cysteine. AIPs are synthesized in the form of pre-peptides which, after posttranslational modification, are released from the cell via the ABC-type transport system. After reaching the threshold concentration in the environment, the AIP molecules are bound by sensor proteins with kinase activity. The kinase is activated by a series of auto-phosphorylation steps followed by the transfer of the phosphate group to a response regulator which can act as a transcriptional regulator of target genes [91]. The best understood example of AIP-mediated QS is the accessory gene regulator (Agr) QS system in *Staphylococcus aureus* [92], which is also present in other Gram-positive bacteria. The autoinducing peptide-based Agr system has been evidenced in some anaerobic rods, as well [39,93,94]. The prototypical *S. aureus* Agr system is composed of a gene operon (*agrACDB*), two promoters P2 and P3, and RNAIII (Figure 7) *agrACDB* encoding proteins:

- cytosolic response regulator acts as a transcription factor (AgrA),
- histidine kinase cell surface receptor (AgrC),
- ~45 residue pro-peptide precursor of the mature AIP (AgrD),
- transmembrane cysteine endopeptidase (AgrB) [39].

The Agr system in *S. aureus* plays a role in the cross-regulation of various proteins, such as immunoglobulin and fibronectin-binding proteins; exotoxins, including hemolysins and enterotoxins; leukocidins; and toxic shock syndrome toxin (TSST). It also regulates enzyme production and promotes the production of phenol soluble modulins (PSMs), amphipathic peptides exhibiting cytolytic activity similar to delta-toxin that participate in many infection-related processes, including the host cells cytolysis and the biofilms dissemination [95,96]. In vitro study conducted by Podkowik et al. demonstrated that the Agr system in *S. aureus* provides long-lived protection from the lethal activity of exogenous H_2_O_2_. The researchers noted that Agr systems found in other bacterial species differ from the *S. aureus*, albeit the relationship between Agr and pathogenesis in most organisms remains unclear [97]. Among anaerobes, Agr systems have been described in *C. difficile*, *C. perfringens*, *Clostridium botulinum*, and *Clostridium tetani*.

Virulence factors for *C. difficile* include sporulation, toxins (enterotoxin A; TcdA, and cytotoxin; TcdB) production, enzymes (collagenase, hyaluronidase, chondroitin sulfatase) releasing, and ability to biofilm formation. The capacity to form biofilms is a crucial factor linked with recurrent *C. difficile* infection (R-CDI) [86,98]. To date, three Agr systems—Agr1, Agr2, and Agr3—have been identified in *C. difficile*, each of which has a different type of gene organization [86]. *C. difficile* Agr2 is similar to *S. aureus*, with genes (*agrACDB*) in reverse order to those found in *S. aureus* [39]. Agr2 is a regulator of flagella formation (regulates bacterial flagella gene expression *fliC, fliA/sidD, fliM*), and TcdA production.

Agr1 system includes the genes for AgrB1 protease and AgrD1 peptide, only. The absence of AgrC1 and AgrA1 suggests that the receptor histidine kinase and transcription factor are encoded in other parts of the genome, or that Agr1 functions in a way that is not strictly dependent on these proteins [39,99]. AgrA or AgrC orthologues forming a two-component signal transduction system are not found in *C. perfringens*, *C. botulinum*, and *Clostridium sporogenes*, as well [39,100]. In the case of *C. difficile*, Agr1 acts as an AIP signaling system in sporulation and plays a role in toxins production, which was confirmed in vivo [101].

**Figure 8:**
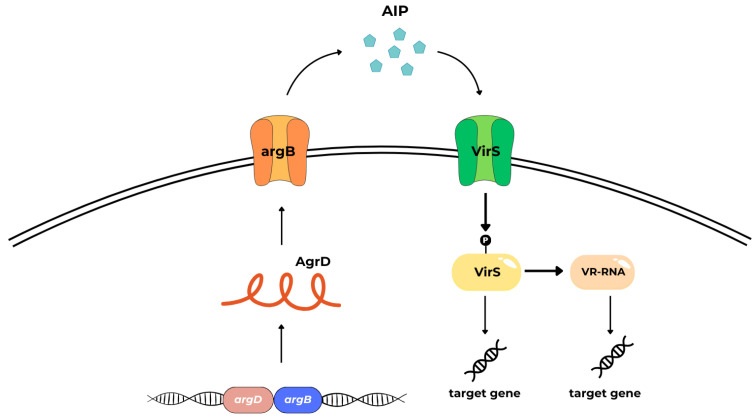
The molecular organization and co-transduction cascade of AgrBD and VirS/R in *C. perfringens*.

Agr3 system consists of AgrB3, AgrD3, and AgrC3 and appears to be encoded by a *C. difficile* bacteriophage (phiCDHM1), suggesting that may be highly mobile and transferred between different *C. difficile* strains. Its function and regulatory mechanisms are poorly understood [39,102]. In *C. difficile* and *C. perfringens*, two functional QS systems are described, the aforementioned LuxS-dependent AI-2 system, which involves the LuxS enzyme, and Agr-like systems [69,82,86,103]. The pathogenicity of *C. perfringens*, a bacterium that causes histotoxicity and enteritis in humans and other mammals, is linked to its ability to produce toxins (more than 20) and extracellular enzymes. Considering the set of toxins produced by *C. perfringens*, seven toxinotypes (from A to G) have been distinguished [104]. In *C. perfringens*, toxin production is controlled by the two-component VirS (membrane sensor protein histidine kinase)/VirR (a response regulator) signal transduction system (TCSTS) and the Agr system. The VirS/VirR system may function similarly to AgrA/AgrC (Figure 8) [105].

The VirS/VirR-VR-RNA cascade controls the pathogenesis by positively regulating virulence related and involved in energy metabolism genes. The study by Mehdizadeh Gohari et al. demonstrates that the VirS protein is a receptor for the AgrD-derived signaling peptide (SP) and that the second extracellular loop of VirS is critical for SP binding. VirS/R is activated by a signal that induces autophosphorylation of VirS, followed by phosphotransfer to VirR. Phosphorylated VirR directly regulates the expression of *netB* (encoding necrotic enteritis B-like toxin, NetB) and *pfoA* (encoding the perfringolysin O, PFO; theta-toxin). In addition, VirS/VirR TCSTS indirectly regulates *cpb* and *cpa* gene expression and thus controls the *C. perfringens* alpha (CPA) and beta toxin (CPB) indirectly by activating the production of a small regulatory RNA called VR-RNA. The VirS is proposed to be the signaling peptide receptor for the *C. perfringens* Agr-like QS system [104,106,107,108].

The Agr-like QS system, which encodes the AgrB and AgrD, is also a virulence regulator because it positively regulates the production of PFO, CPA, CPB, and NetB, sporulation, and *C. perfringens* enterotoxin (CPE) production (toxinotype F) [100,103,105,109,110]. Seven immunologically distinct botulin neurotoxins (BoNTs) designated by the letters from A to G and more than forty different subtypes are produced by six phylogenetically distinct clostridia (*C. botulinum* groups I–IV and some strains of *C. baratii* and *C. butyricum*). Toxigenic strains are associated with severe flaccid paralysis in vertebrates. Pathogenicity of *C. botulinum* is controlled by the QS system; however, regulation of botulinum neurotoxin gene (*bont*) expression and BoNT production are not fully understood [111]. The Agr system (Agr-1/Agr-2) has been evidenced in *C. botulinum* strains classified into group I. Agr-1 appears to be involved in sporulation, while Agr-2 is in neurotoxin production. Agr-1 and Agr-2 are homologs of AgrB, and AgrD proteins in *C. perfringens* [98].

## 5. Prevention of Biofilm Formation by Quorum Quenching (QQ) and Perspectives of QQ Application

In this review, we discuss the QS mechanisms that have been described so far in anaerobic bacteria to present potential solutions affecting the possibility of QS disruption. The process of interfering with microbial cell-to-cell communication is referred to as quorum quenching. QQ inhibition can be achieved in a variety of ways. Proposed QQ strategies in anaerobic bacteria include the following:

- inactivation or enzymatic degradation of signaling molecules.
- competition with signaling molecules; competing with inducers for the same binding site or binding the receptor noncompetitively.
- blocking of signal transduction cascades.

The best studied QQ mechanism is signal degradation by enzymatic pathways or by means of analogs of signal molecules.

### 5.1. Inhibitors of AHL-Mediated Quorum Sensing

The chemical structure of AHLs suggests that the degradation of these molecules may be mediated by lactonases, acylases, decarboxylases, deaminases, and oxidoreductases [54].

The most extensively studied groups of AHL-degrading enzymes are *N*-acyl-homoserine lactonases (AHL-lactonases) and N-acyl-homoserine lactone acylase (AHL-acylases). AHL-lactonases degrade AHL by hydrolyzing the lactone ring in the homoserine moiety of AHLs, while AHL-acylases hydrolyze the amide bond between the acyl side chain and the homoserine lactone in the AHL molecules producing the free fatty acid and the homoserine lactone [51,112]. One of the first described and best analyzed AHL lactonases is AiiA_24B1_, the enzyme produced by *Bacillus* sp. 24B1 *aiiA* gene [113]. Kinetic and substrate specificity analyses showed that AHL-lactonase had little or no residual activity towards non-acyl lactones and non-cyclic esters but showed strong enzyme activity towards many of the AHLs evaluated, regardless of the length and type of substitution at the C3 position of the acyl chain. AHL lactonases are expressed in most pathogens [113]. In the study conducted by Muras et al., the AHL-lactonase Aii20J caused a significant reduction in oral biofilms growing under aerobic and anaerobic conditions in vitro. Confocal microscopy analysis of in vitro multi-species oral biofilms formed by *A. naeslundii*, *A. actinomycetemcomitans*, *F. nucleatum*, *P. gingivalis*, *S. oralis*, and *Veillonella parvula* revealed a significant inhibition of biofilms formation when Aii20J was added to the culture media [51]. Parga and co-workers showed that Aii20J modulates polymicrobial biofilm formation without altering the microbiome structure of the biofilm [114]. Murugayah’s team using a fluorescamine-based assay demonstrated that AHL-acylase can degrade quorum-sensing molecules [51,61]. AHL acylase activity has been identified in several bacterial species. Their substrate specificity is based on the different acyl chain substitutions of AHLs. It has been proposed that acylases degrade AHL with a long side chain more than those with a short side chain [115].

**Figure 9:**
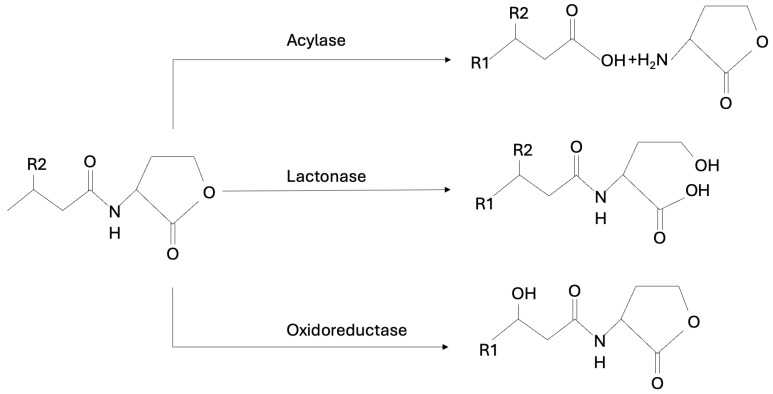
AHL-degrading enzymes as an AHL-mediated inhibitor.

There are also several known AHL oxidoreductases found in different bacteria that can reduce or oxidize the acyl chain of AHL, thereby inhibiting the specific binding of the autoinducer to its receptor. AHL oxidoreductases are modifying enzymes classified into two groups: reductases, which can convert 3-oxo-substituted AHLs to 3-hydroxyl AHLs, and cytochrome oxidases, which catalyze the oxidation of the acyl chain. The modified compounds can no longer function as signaling molecules without being degraded [115]. It has been evidenced that some bacteria produce substantial amounts of AHL-lactonase, acylase, or oxidoreductase enzymes. The enzymatic pathway of AHL-inhibitors is shown in Figure 9. Enzymatic QQ is the best studied QS inhibition strategy and is an interesting alternative to the problem of bacterial resistance to antibiotics. The potential of these enzymes depends on the quantity of other enzymes, their level of activity, biotechnological possibilities of synthesis, and stability [116].

Another strategy relevant to QQ is to inhibit the synthesis of the AHL signaling molecule through the use of signaling molecule analogs [62]. AHL-analogs modify not only the protein expression but also slow down the growth of *P. gingivalis*. This finding potentially opens new perspectives for the prevention or treatment of periodontal disease [51]. Blocking the LuxR/AHL interaction is most often caused by molecules that are AHL antagonists competing with signaling molecules for a binding site on the receptor [117].

### 5.2. Inhibitors of AI-2-Mediated Quorum Sensing

The quenching of signal molecule activity can be achieved by AI-2 quorum-sensing inhibitors (QSIs), which include AI-2 analogs. AI-2 mediates both intra- and inter-species communication and is considered notably a potential target for the control of periodontal diseases. It has been shown that monosaccharides, including D-ribose and D-galactose, reduce virulence gene expression and biofilm formation by blocking the AI-2 receptor, hence they could potentially be used for the prevention of the biofilm formation of periodontopathogens [54,65,70,79].

Ribose, as furanosyl borate diether, is structurally similar to AI-2 and has minimal toxic side effects. D-galactose significantly reduced the biofilm formation of *F. nucleatum*, *P. gingivalis,* and *T. forsythia* induced by AI-2 of *F. nucleatum* [65]. An et al. demonstrated that D-arabinose significantly reduces AI-2 activity and biofilm formation of oral bacteria (*S. oralis*, *F. nucleatum*, and *P. gingivalis*) on titanium discs. The researchers suggested that L-arabinose has initial anti-adhesive activity, as well [79].

Coumarins isolated from *Coumarouna odorata* (heterocycles, consisting of a benzene ring linked to a pyrone ring) are a group of compounds with potential antimicrobial utility as well. Coumarins are a class of bioactive compounds present in many plant sources, including beans, sweet clover, cinnamon oil, and lavender products. Their antiviral, antimicrobial, anti-inflammatory, antitumor, antioxidant, and anticoagulant activity are well known [13,118]. It has been shown that the incorporation of another heterocyclic group into the coumarin molecule can enrich the biological properties of the matrix compound. To date, coumarin derivatives have been reported to inhibit biofilm formation in *S. aureus*, *Escherichia coli*, and *Chromobacterium violaceum* [119]. He et al. demonstrated that coumarin at sub-MIC concentrations, without affecting bacterial growth, inhibited *P. gingivalis* biofilm formation. Anti-biofilm effects for the late-stage and pre-formed biofilm dispersion were also documented. After coumarin treatment, the biofilms became interspersed. Coumarin inhibited *P. gingivalis* biofilm formation through a QS system by interacting with the heme-binding protein (HmuY) that plays a leading role in *P. gingivalis* heme acquisition [13].

The study conducted by Marquis et al. confirmed that the growth of *P. gingivalis* was inhibited when exposed to the lacinartin compound, natural oxyprenylated coumarin present in the culture medium. Lacinartin also inhibited *P. gingivalis* biofilm formation, enhanced biofilm detachment, prevented *P. gingivalis* adherence to oral epithelial cells, inhibited *P. gingivalis* collagenase activity, reduced the secretion of cytokines (IL-8 and TNF-α) and matrix metalloproteinases (MMP-8 and MMP-9) by LPS-stimulated macrophages, and inhibited MMP-9 activity [120]. The molecular mechanism of lacinartin has not been explained.

Produced by the macroalga *Delisea pulchra*, brominated furanones are reported to inactivate (by covalently modifying) the LuxS enzyme, required for AI-2 synthesis, thereby inhibiting the QS activity of various bacterial species. The bromofuranone analog, 3-(dibromomethylene) isobenzofuran-1(3H)-one derivative demonstrated inhibitory activities against biofilm formation by periodontopathogens (*F. nucleatum*, *P. gingivalis*, and *T. forsythia)* without a bactericidal effect [70,121].

Heparinoids (glycosaminoglycans) are chemically and pharmacologically related to heparin, known for its anticoagulant activity. Heparinoids suppress *C. acnes* biofilm formation via AI-2 inhibition. The study conducted by Hamada et al. showed that heparinoids at low concentrations may lead to decreased lipase activity that is associated with irritation and inflammation specific to acne. The authors also demonstrated that heparinoids enhance isopropyl methylphenol (IPMP) bactericidal efficacy against *C. acnes* biofilms [122].

Phlorizin and phloretin polyphenols are commonly found in many types of human diets and possess anti-biofilm properties against *P. gingivalis.* Phloretin is a chalcone flavonoid naturally occurring in apple fruit, bark, and leaves. Phlorizin, a derivative of phloretin, has an additional glycoside in its structure [123].

Phloretin can inhibit the biofilm formation of the oral pathogen *Streptococcus mutans* and has the capability to suppress its QS by inhibition of glucosyltransferases GtfB and GtfC (enzymes that split sucrose into glucose and fructose and link the glucose moiety together via glycosidic bonds to form EPSs) [124]. In *S. mutans*, the signaling transduction system (VicRK) positively regulates the expression of *gtfB/C* genes by binding their promoter regions [125]. Genes encoding *P. gingivalis* glycosyltransferases involved in O-LPS and A-LPS biosynthesis, named *gtfC*, *gtfD*, *gtfE*, and *gtfF*, are identified by Shoji et al. [124]. Scanning electron microscopy showed that phloretin and phlorizin displayed a similar and remarkable destructive effect on *P. gingivalis* and the mixed biofilms [123]. This aligns with findings from transcriptome analysis conducted by Wu et al. confirmed that phlorizin and phloretin reduced AI-2 activity to 45.9% and 55.4%, which means that they can interfere with *P. gingivalis’* intercellular communication. Furthermore, while other flavonoids with similar structures, such as naringenin, have been reported to inhibit the growth of *P. gingivalis*, *F. nucleatum*, and *S. mitis*, there are no studies specifically addressing their effects on biofilm inhibition [123].

Xu and co-workers have shown that reuterin (isolated from *Lactobacillus reuteri* LR 21) significantly suppressed the biofilm formation of *C. perfringens*. As mentioned earlier, toxin production and pathogenicity of *C. perfringens* depended on the Agr and LuxS quorum-sensing system. The authors hypothesized that the downregulation of *agrB* and *luxS* in *C. perfringens* treated with reuterin appears to be responsible for the decreased expression levels of *cpa* and *pfo* genes [126].

### 5.3. Inhibitors of Agr-like Quorum Sensing

It has been shown that in *C. perfringens*, an Agr-like QS system through a VirS/R two-component signal transduction system regulates the expression of virulence genes. Based on the structure-activity relationship (SAR) data on 5-residue thiolactone peptide AIP, two inhibitory peptides designed to target VirS, the receptor histidine kinase AIP, were found to attenuate Agr-mediated toxin production. One of them is a partial agonist (Z-AIP_Cp_-L2A/T5A), the second is a partial antagonist (Z-AIP_Cp_-F4A/T5S). To determine the agonist/antagonist activity of synthetic peptides, a transcriptional response was monitored by quantifying the level of *pfoA* after *C. perfringens* incubation with the tested peptides. In a virulent strain, both peptides significantly attenuated the transcription of the theta-toxin gene (*pfoA*) [127].

Inhibitors that have the potential to disrupt QS in anaerobic bacteria are demonstrated in Table 2.

## 6. Summary, Constraints, and Prospects

Biofilms are a critical survival strategy for pathogenic bacteria, serving as both non-specific virulence factors and non-specific mechanisms of antibiotic resistance. Quorum sensing, as a type of microbial cell-to-cell communication system, plays a key role in the biofilm formation process at every stage of development. The phenomenon of QS was discovered over five decades ago with the observation that bioluminescence in the marine bacterium *Photobacterium fischeri* only occurred at increased cell densities.

In anaerobic bacteria, QS rely on certain molecules known as autoinducers, such as AHLs (AI-1), AI-2s, or AIPs. Among these, the AI-2-dependent mechanism seems to be particularly important for this group of microorganisms. Several periodontal bacteria, such as *P. gingivalis*, *T. denticola*, *P. intermedia, F. nucleatum*, and *C. difficile*, associated with post-antibiotic diarrhea, have been studied for their QS capabilities, making them potential targets for quorum quenching strategies [46,86].

The quorum-quenching strategy may be a promising target for the development of a novel non-antibiotic therapy. Despite its evident potential, several challenges remain. The effectiveness of QQ strategies can vary significantly due to the diversity of microbial communities and the complexity of biofilm structures. Future research should focus on understanding the mechanisms of QQ in different environments, exploring its synergy with other antimicrobial strategies, and developing targeted QQ applications. Ongoing advances in biotechnology may pave the way for innovative solutions to biofilm-related problems, making QQ an exciting area of study in microbial ecology and applied sciences.

In conclusion, quorum quenching represents a novel approach to preventing biofilm formation. By disrupting bacterial communication, QQ strategies could transform the way we tackle the challenges posed by biofilms in medicine and across many other industries, leading to progress towards safer medical practices, better food preservation, and maintaining a cleaner environment.

## Figures and Tables

**Table 1:** Influence of AI-2 signaling molecules on phenomena occurring in biofilms formed by anaerobic bacteria.

Biological Processes Associated with AI-2 in Biofilm Formed by Anaerobic Bacteria	References
LuxS signaling in *P. gingivalis* plays a significant role in interaction with fibroblasts. This may be through the downregulation of the gene PGN_0482, which encodes a putative outer membrane immunoreactive protein.	[47]
LuxS/AI-2 signaling mediates the interaction between *P. gingivalis* and periodontitis-associated species like *Streptococcus gordonii.*	[77]
The *luxS* gene from *A. actinomycetemcomitans* can complement a *luxS* mutation in *P. gingivalis.*	[84]
LuxS/AI-2 signaling in *P. gingivalis* regulates hemin acquisition, growth in hemin-limited conditions, and the expression of proteases and stress-related genes. In Δ*luxS* strains, genes encoding hemin binding protein (*tlr*) and (lysine-specific protease) *kgp* genes are downregulated, while gene *hmuR* encoding an outer-membrane hemin utilization receptor, *fetB encoding* heme-binding protein, *feoB1 encoding* ferrous iron transporter, and *ftn* ferritin-like proteins are upregulated.	[74,76]
LuxS signaling is involved in promoting the survival of *P. gingivalis* in the host by regulating its response to host-induced stress factors (H_2_O_2_, and high pH). In the *luxS* mutant, genes related to stress response (*htrA*, *clpB*, *groEL*, *dnaK*, and ahpF), coding outer membrane efflux protein, and immunoreactive antigen are upregulated.	[78]
In *P. gingivalis*, LuxS modulates protease levels and hemagglutination. *luxS* mutants exhibited a reduction in haemagglutinin titer and lower Rgp and Kgp proteases (gingipains) activity.	[75]
AI-2 from *F. nucleatum* enhanced single species biofilm formation of *F. nucleatum*, *P. gingivalis, T. denticola,* and *T. forsythia*, and promoted co-aggregation with red complex species (*P. gingivalis*, *T. denticola*, *T. forsythia*). AI-2 induces mRNA synthesis of adhesion molecules like FadA, RgpA, Msp, and BspA (representative adhesion molecules of these bacteria).	[70]
*F. nucleatum* AI-2 triggers inflammatory responses and promotes macrophage mobility and M1 polarization via the TNFSF9/TRAF1/p-AKT/IL-1β pathway.	[67,72]
AI-2 may induce prophages in *C. difficile* biofilms, leading to phage-mediated cell lysis and eDNA release, enhancing biofilm growth.	[69]
*C. diffcile* LuxS-mediated prophage induction. LuxS deficiency in *C. difficile* impairs prophage induction and biofilm formation in vitro.	[85,86]
*C. perfringens* AI-2 regulates toxin production. *luxS* activates *pfoA* transcription and theta-toxin production and possibly influences post-transcriptional regulation of the production of alpha and kappa toxins.	[82]
In *C. acnes*, AI-2 enhances virulence by increasing bacterial lipase activity.	[83,87]
*C. difficile* AI-2 in co-culture triggers selective metabolic responses in *B. fragilis*, downregulating carbon metabolism genes; genes related to alanine, aspartate, and glutamate metabolism; and involved in amino acid biosynthesis.	[69,88]
*LuxS* mutants of *Lactobacillus rhamnosus* GG and *Bifidobacterium breve* UCC2003 are less persistent in the murine gastrointestinal tract than wild strains, likely due to increased sensitivity to gastric fluid and impaired iron acquisition.	[89,90]

**Table 2:** Review of proven QQ strategies in pathogenic anaerobic bacteria.

Potential QQ Inhibitors	Refs.
**Inhibitors of AHL-Mediated Quorum Sensing**	
AHL-degrading enzymes AHL-degrading enzymes are lactonases, acylases, and oxidoreductases. AHL-lactonase inhibits the QS process by hydrolyzing the lactone ring in the homoserine moiety of AHLs.	[51,62,112,115,116,117]
AHL-acylases hydrolyze the amide bond between the acyl side chain and the homoserine lactone in the AHL molecules producing the free fatty acid and the homoserine lactone.	
AHL oxidoreductases reduce or oxidize the acyl chain of AHLs.	
AHL analogs AHL-analogs inhibit AHL synthesis, alter protein expression, and slow the growth of *P. gingivalis*, offering the potential for periodontal disease treatment.	
AHL antagonists AHL antagonists compete with natural signaling molecules for receptor binding by blocking the interaction between LuxR and AHL and disrupting quorum sensing.	
**Inhibitors of AI-2-mediated quorum sensing**	
AI-2 analogs AI-2 analogs are D-ribose and D-galactose. These monosaccharides can block AI-2 receptors, reducing virulence gene expression and biofilm formation of periodontopathogens including *A. actinomycetemcomitans*, *F. nucleatum*, *P. gingivalis*, *T. forsythia*, and *T. denticola*. The production of bacterial adhesins was markedly reduced when the bacteria were grown in the presence of D-ribose. D-galactose reduces biofilm formation of *F. nucleatum*, *P. gingivalis*, and *T. forsythia* by blocking the AI-2 receptor.	[65,70,79]
D-arabinose reduces biofilm formation by potentially competing with salivary receptors or bacterial adhesins.	
Coumarin Coumarin inhibits biofilm formation by *P. gingivalis* through interaction with the heme-binding protein HmuY and causes its interspersal and dispersion in pre-formed and late-stages.	[13,118,119]
Lacinartin Lacinartin inhibits *P. gingivalis* growth, biofilm formation, and collagenase activity. Prevents adherence to oral epithelial cells and reduces inflammatory responses—secretion of IL-8 and TNF-α and matrix metalloproteinases (MMP-8 and MMP-9).	[120]
Brominated furanones Brominated furanones inactivate the LuxS enzyme, required for AI-2 synthesis. The bromofuranone analog, 3-(dibromomethylene) isobenzofuran-1(3H)-one derivative inhibits biofilm formation by *F. nucleatum*, *P. gingivalis*, and *T. forsythia* without killing the bacteria.	[70,121]
Heparinoids Glycosaminoglycans inhibit AI-2 activity, thereby reducing biofilm formation and lipase activity in *C. acnes*, additionally enhancing the bactericidal effects of isopropyl methylphenol on *C. acnes* biofilms.	[122]
Phlorizin and phloretin polyphenols Phlorizin and phloretin disrupt biofilm forming by *P. gingivalis* and suppress its QS by inhibition of glucosyltransferases GtfB and GtfC.	[123,124,125]
Reuterin Reuterin, produced by *L. reuteri*, suppresses biofilm formation of *C. perfringens* and downregulated QS-related genes like *agrB* and *luxS*, which decreases toxin production.	[126]
**Inhibitors of Agr-like quorum sensing**	
In *C. perfringens*, a partial agonist (Z-AIPCp-L2A/T5A) and a partial antagonist (Z-AIPCp-F4A/T5S), significantly reduces transcription of the theta-toxin gene (*pfoA*).	[127]
